# Granulomatous Sarcoidosis Mimics

**DOI:** 10.3389/fmed.2021.680989

**Published:** 2021-07-08

**Authors:** Marc A. Judson

**Affiliations:** Division of Pulmonary and Critical Care Medicine MC-91, Department of Medicine, Albany, NY, United States

**Keywords:** sarcoidosis, diagnosis, mimics, granuloma, drug reaction, infection, vasculitis

## Abstract

Many granulomatous diseases can mimic sarcoidosis histologically and in terms of their clinical features. These mimics include infectious granulomatous diseases, granulomatous reactions to occupational and environmental exposures, granulomatous drug reactions, vasculitides and idiopathic granulomatous conditions. It is important to distinguish sarcoidosis from these mimics, as a misdiagnosis of these diseases may have serious consequences. This manuscript reviews numerous sarcoidosis mimics and describes features of these diseases that may allow them to be differentiated from sarcoidosis. Distinguishing features between sarcoidosis and its mimics requires a careful review of the medical history, symptoms, demographics, radiographic findings, histologic features, and additional laboratory data. Understanding the clinical characteristics of sarcoidosis and its mimics should lead to more accurate diagnoses and treatment of granulomatous disorders that should improve the care of these patients. As the diagnostic criteria of sarcoidosis are not standardized, it is possible that some of these sarcoidosis mimics may represent varied clinical presentations of sarcoidosis itself.

## Introduction

Sarcoidosis is a multisystem granulomatous disease of unknown cause. The diagnosis of sarcoidosis is arbitrary (1), not standardized (2), and is never completely secure (2). Because of the inexactness of the diagnostic criteria for sarcoidosis, alternative conditions with similar clinical features to sarcoidosis may be misdiagnosed as sarcoidosis. These “sarcoidosis mimics” are important to identify, as misdiagnosing them as sarcoidosis and treating them inappropriately may have significant health consequences. In this manuscript, we will review the clinical manifestations of various sarcoidosis mimics and describe their distinguishing features from sarcoidosis.

For the purposes of this discussion, we will discuss only granulomatous diseases that mimic sarcoidosis. We will not address diseases that may mimic sarcoidosis in terms of presenting symptoms (e.g., a systemic illness), radiographic findings (e.g., bilateral hilar adenopathy on chest radiograph) or laboratory findings (e.g., elevated serum angiotensin converting enzyme level) that are not associated with granulomatous inflammation.

## Problems With The Diagnosis of Sarcoidosis That Affect The Definition of A Sarcoidosis Mimic

The diagnosis of sarcoidosis is based on three major criteria: a compatible clinical presentation, identifying non-necrotizing granulomatous inflammation in one or more tissues, and the exclusion of alternative causes of granulomatous disease (2). Currently, no objective measures have been established to confirm any of these three criteria (2). The compatible clinical presentation and exclusion of alternative causes of granulomatous disease criteria essentially rely on the clinician's judgment. There is also controversy as to whether one or more than one organ must have evidence of granulomatous inflammation in order to establish the diagnosis of sarcoidosis (3). The presence of granulomas in more than one organ would fulfill the requirement that the disease is “systemic.” However, many other features of sarcoidosis besides multi-organ involvement can establish that the disease is systemic, including its association with anergy, polyclonal gammopathy, specific inflammatory syndromes (e.g., erythema nodosum) (4), and the recurrence of sarcoidosis in allografts of organ transplant recipients with sarcoidosis (5). The contention that multiple organ involvement is not required to establish a diagnosis of sarcoidosis is highlighted by the fact that in the National Institute of Health A Case Control Etiology of Sarcoidosis Study (ACCESS), half of the sarcoidosis cohort had evidence of sarcoidosis in only one organ (6). To complicate matters further, at times the diagnosis of sarcoidosis can be established on the basis of clinical features that are highly specific for the diagnosis without performing a tissue biopsy (2, 7).

Sarcoidosis is associated with many different occupational and environmental exposures (8) and has a wide variety of phenotypic expressions (9). Since the diagnosis of sarcoidosis is inexact, it is unclear whether these different associated exposures and phenotypes represent distinct diseases [various “sarcoidoses” (10)] or are different presentations of sarcoidosis. To that end, the line of demarcation between sarcoidosis and a mimic of the disease may be blurred. “Lumpers” may favor casting a broad net and classify various forms of sarcoidosis-like diseases as sarcoidosis, whereas “splitters” may prefer differentiating sarcoidosis from similar conditions that fail to meet certain pre-set requirements. Because of the blurring of the boundary between what is classified as sarcoidosis and what is not, we acknowledge that some of the sarcoidosis mimics that we will discuss may, in actuality, be variant forms of sarcoidosis. Although sarcoidosis is a multisystem disease of unknown cause, it is obvious that sarcoidosis does have a cause. In the future when the cause of sarcoidosis is elucidated, it will become clear which conditions constitute a form of sarcoidosis and which are sarcoidosis mimics.

## The Range of Granulomatous Sarcoidosis Mimics

Table 1 lists various conditions that are potential granulomatous mimics of sarcoidosis. These conditions include infections, malignancies, vasculitides, inflammatory responses to environmental and occupational exposures and idiopathic inflammatory responses. These mimics should be considered as potential alternative diagnoses to sarcoidosis, as inappropriate anti-sarcoidosis treatment may worsen several of these conditions, result in unnecessary drug toxicity, and inadequately treat the actual disease. We discuss several of these granulomatous sarcoidosis mimics in detail in the remainder of this manuscript.

**Table 1:** Potential granulomatous mimics of sarcoidosis.

**General categories**	**Specific conditions**
**Infections**	
Mycobacteria	Tuberculosis
	Non-tuberculous mycobacteria
Fungi	Cryptococcus
	Histoplasmosis
	Blastomycosis
	Coccidioidomycosis
	Aspergillosis
Other infections	Mycoplasma
	Pneumocystis jiroveci
	Brucellosis
	Toxoplamosis
	Leishmaniasis
	Schistosomiasis
	Bartonella
	Mononucleosis (Epstein Barr virus)
	Cytomegalovirus
	Coxiella burnetii (Q fever)
	Treponema (syphilis, yaws)
**Environmental and occupational exposures**	
Hypersensitivity pneumonitis	
Pneumoconioses	Beryllium (chronic beryllium disease)
	Titanium
	Aluminum
Malignancies	Lymphoma
	Sarcoidosis-like reaction of malignancy
Vasculitidies/Connective tissue diseases	Granulomatosis with polyangiitis
	Rheumatoid nodules
Localized granulomatous reactions to foreign substances	Lung aspiration
	Foreign body reactions
Drug-induced sarcoidosis-like reactions (DISRs)	Highly active retroviral therapy (HAART)
	Immune checkpoint inhibitors
	Tumor necrosis alpha antagonists
	Interferon
	Other drugs
Diffuse granulomatous reactions from an autoimmune inflammatory syndrome induced by adjuvants	
Granulomatous lesions of unknown significance (GLUS syndrome)	
Granulomatous interstitial lung disease (GLILD) related to common variable immunodeficiency (CVID)	
Necrotizing sarcoid granulomatosis	
Blau syndrome	
Orofacial granulomatosis	
Crohn's disease	
Primary biliary cirrhosis	

**Figure 1:**
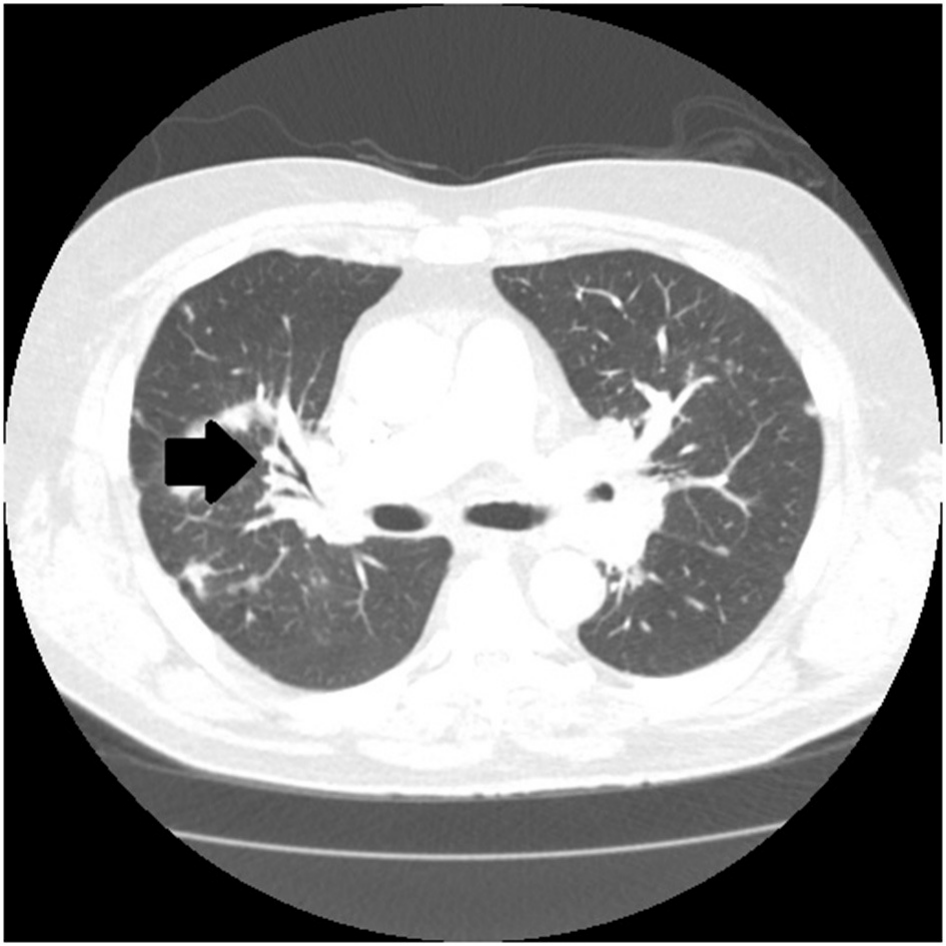
Chest CT scan showing sarcoidosis nodules coalescing around the bronchovascular bundle (arrow).

**Figure 2:**
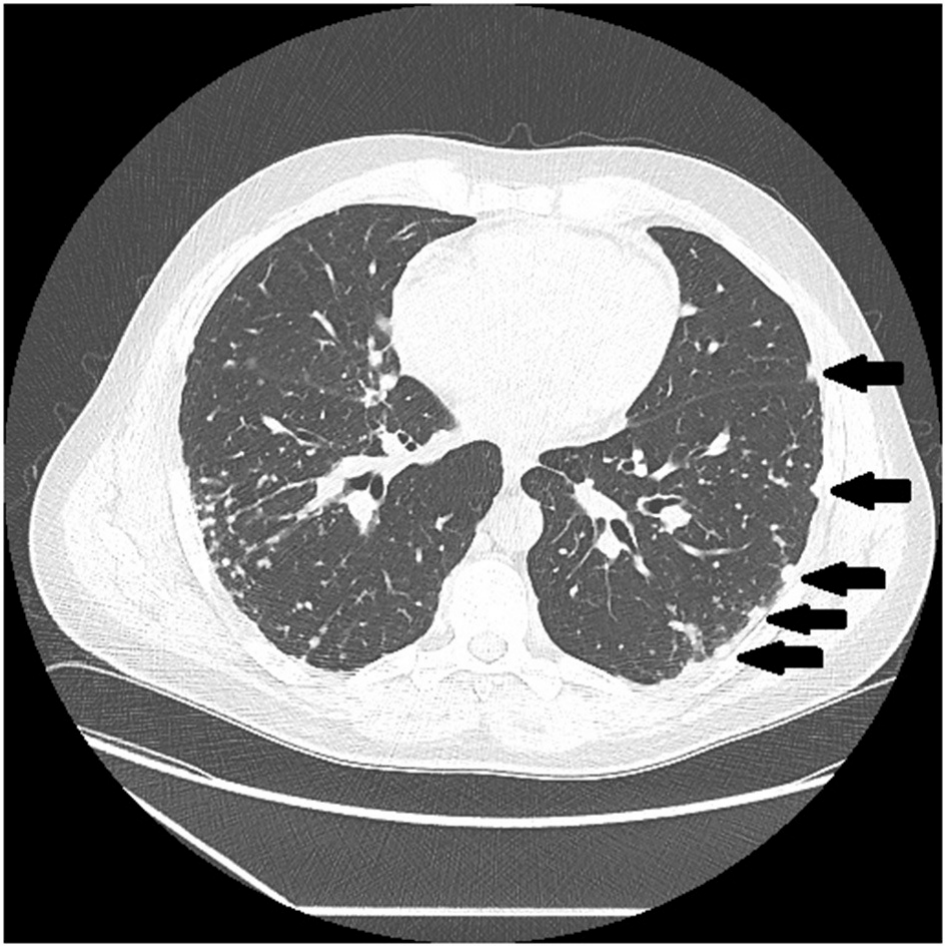
Chest CT scan showing multiple subpleural nodules from sarcoidosis (arrows).

**Figure 3:**
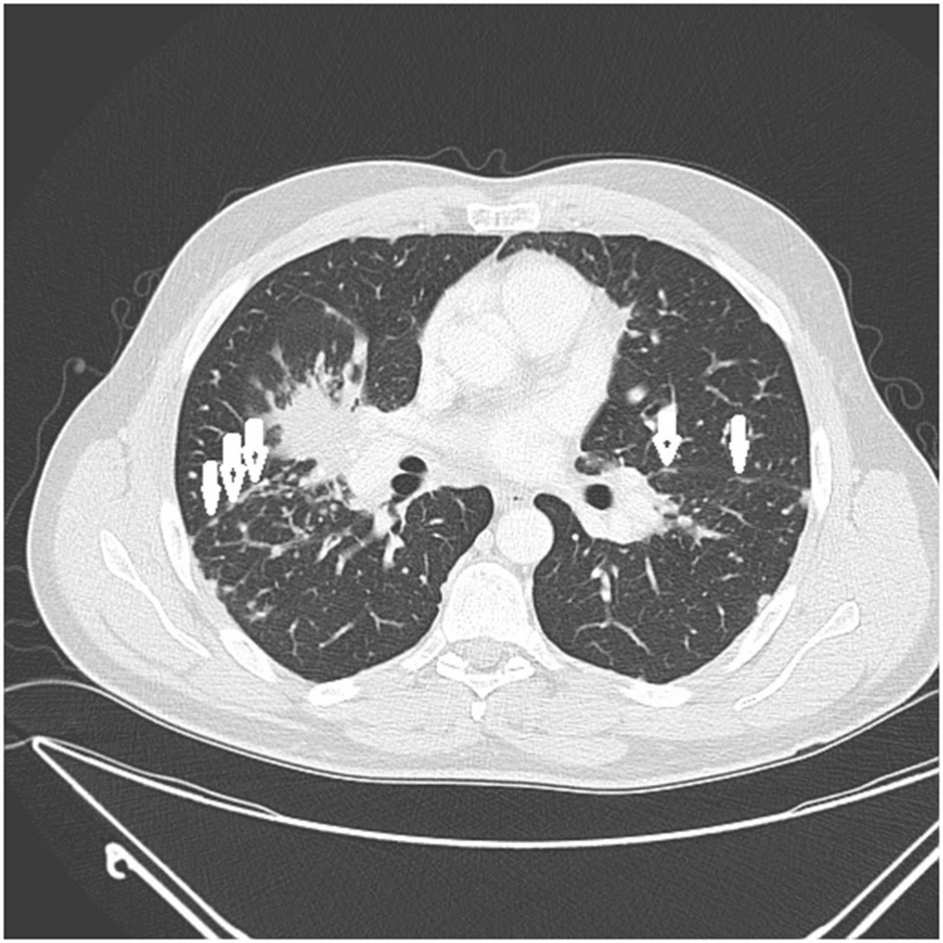
CT scan showing beading of the left and right major fissures with sarcoid granulomas (arrows).

**Figure 4:**
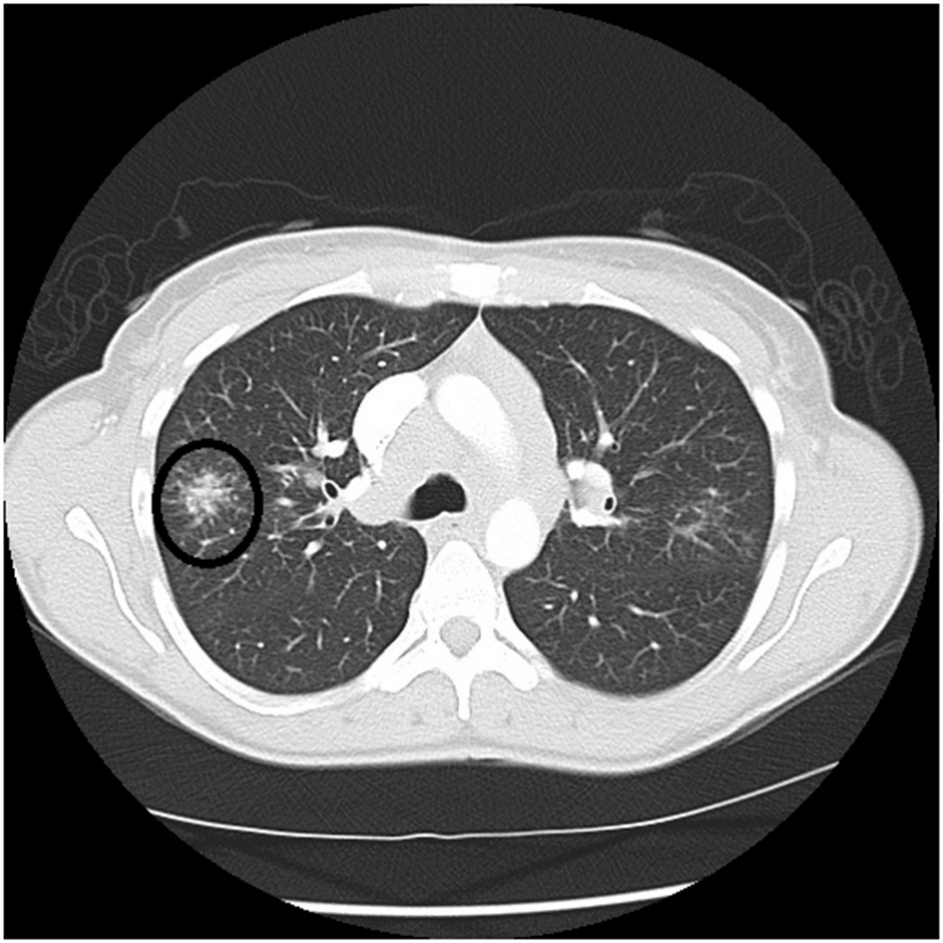
Chest CT scan showing a galaxy sign in sarcoidosis, where micronodules coalesce centrally into a mass lesion (circle).

**Figure 5:**
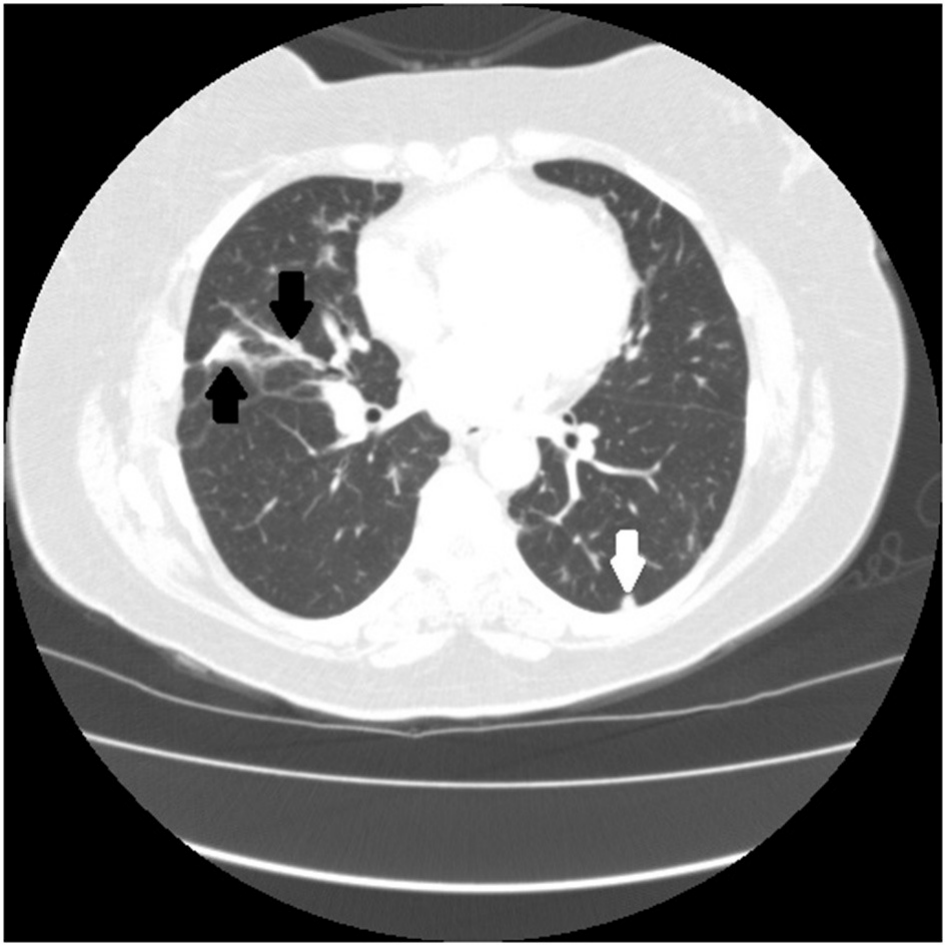
Chest CT scan of granulomatous-lymphocytic interstitial lung disease from common variable immunodeficiency. This condition may have identical radiographic and histologic findings of sarcoidosis. Nodules are demonstrated along the bronchovascular bundle (black arrows) and a subpleural nodule (white arrow) that are commonly observed with sarcoidosis (see Figures 1, 2).

There are certain radiographic features that are typical for sarcoidosis, and these are listed in **Table 3**. Figures 1–4 provide examples of some of these radiographic findings. However, it is important to note that such specific radiographic findings are often not present. In addition, some sarcoidosis mimics may present as radiographic mimics of the disease, and present with identical radiographic findings listed in **Table 3** and Figure 5. For these reasons, distinguishing sarcoidosis from pulmonary granulomatous mimics solely on the basic of radiographic findings is unreliable and generally not recommended.

## Infections

### Mycobacterium Tuberculosis

Numerous infectious human pathogens may elicit a granulomatous response and need to be considered as an alternative diagnosis to sarcoidosis. *Mycobacterium tuberculosis* (MTB) is the most common infectious granulomatous lung disease in the world (11–13). MTB may mimic sarcoidosis, and it is imperative that MTB be reliably excluded before a diagnosis of sarcoidosis can be secured. Patients with MTB infection usually present with insidiously progressive symptoms over weeks to months that include cough, weight loss, fever, night sweats, and fatigue. These symptoms may all be seen with sarcoidosis, as cough is a very common symptom (14) and “constitutional symptoms” of fever, night sweats, weight loss, and fatigue are present in up to one-third of sarcoidosis patients on presentation (15). Although typical radiographic presentations of pulmonary tuberculosis differ from the usual chest imaging patterns of sarcoidosis, there is significant overlap of their radiologic manifestations that they cannot be reliably distinguished on that basis. Typical chest imaging findings of reactivation tuberculosis include focal parenchymal opacities, frequent cavitation, pleural involvement, and endobronchial spread of disease (16). These findings are not common with pulmonary sarcoidosis, but may be seen (17, 18). The radiographic findings of primary tuberculosis include hilar and mediastinal lymphadenopathy, pleural effusion, and a solitary pulmonary nodule that have all been reported in cases of pulmonary sarcoidosis (18–20). The presence of bilateral hilar lymphadenopathy on a chest radiograph without B symptoms (fever, night sweats, weight loss) is extremely specific for sarcoidosis and has been suggested by some as diagnostic for sarcoidosis (2, 21). Histologic features of the granuloma also fail to reliably distinguish tuberculosis from sarcoidosis. Although necrosis is typically observed in granulomas with MTB infection and sarcoidosis granulomas are thought to be non-necrotizing, non-caseating granulomas may be present with MTB (22), and sarcoidosis granulomas have necrotic features in up to one third of cases (23, 24).

Because of the overlap of the symptoms, radiographic features, and histology of sarcoidosis and tuberculosis, MTB infection needs to be excluded in all patients where the diagnosis of sarcoidosis is considered. The process of excluding MTB infection in a potential sarcoidosis patient begins with obtaining an adequate history of a suspected exposure to a person with active pulmonary tuberculosis In addition, in potential sarcoidosis patients who undergo a biopsy to detect granulomatous inflammation, tissue staining and culture for MTB should be performed. Molecular assays such as the probe-based nucleic acid amplification test (25) and sequence based assays such as the Xpert^®^ MTB/RIF (26) may be used as adjunctive tests in selected cases. Table 2 lists various historical, histologic and radiographic features that may distinguish sarcoidosis from tuberculosis.

**Table 2:** Clinical data supporting the possibility of sarcoidosis vs. tuberculosis.

	**Supports sarcoidosis**	**Supports tuberculosis**	**Does not favor either diagnosis**
**Clinical presentation**			
Fever, weight loss, night sweats			√
History of tuberculosis contact		+	
Hemoptysis		+	
Extrapulmonary manifestations (eye disease, skin disease)	+		
**Histology**			
Non-caseating granulomas	+		
Caseating granulomas		+	
Schaumann bodies	+		
Asteroid bodies	+		
Mycobacterial stains positive		++	
**Radiology**			
Cavitation		+	
Upper lobe disease			√
Necrotic mediastinal lymphadenopathy		+	
Non-necrotic mediastinal lymphadenopathy			√
Galaxy sign	++		
Tree-in-bud opacities		+	
Perilymphatic distribution of nodules	++		

*+: supports diagnosis ++: strongly supports diagnosis √: condition common in both disorders*.

### Non-tuberculous Mycobacteria and Fungi

Several other infections are associated with granulomatous inflammation including non-tuberculous mycobacteria (NTM) and various fungi. Because NTM and fungi are common infectious causes of granulomatous inflammation (11, 27, 28), all diagnostic biopsies in sarcoidosis patients should be routinely stained and cultured for these pathogens (29). Not only are the histologic findings of the granulomatous inflammation of MTB and NTM infection indistinguishable, but the slight morphologic differences of the organisms identified by staining are not reliable to separate these organisms (30). Therefore, culture or molecular assays are required to identify the pathogen. Typical histologic findings of NTM show necrotizing granulomas (30), although non-necrotizing lesions may be seen (31). Granulomatous inflammation occurs with histoplasmosis, cryptococcus, blastomycosis, coccidiodomycosis, and less commonly with aspergillus species and other fungi (30). The histology of fungal infections usually demonstrates necrotizing granulomas (30), however, similar to mycobacterial infections, non-necrotizing granulomas are found on occasion (32–35). Fungal infections may be diagnosed by stain and culture of the organism (30), serologic techniques (36), or molecular methods (37, 38). Several other infections may induce a granulomatous response as shown in Table 1, and these may all occasionally be non-necrotizing (39).

## Occupational and Environmental Exposures

### Hypersensitivity Pneumonitis

Various environmental and occupational exposures may cause granulomatous disease. Hypersensitivity pneumonitis (HP) is a granulomatous lung disease induced by a wide variety of organic bioaerosols and airborne chemicals. These aerosols induce an allergic type 3 (antigen-antibody) and type 4 (granulomatous) response within the lung in susceptible individuals. HP has three clinical presentations: acute, subacute and chronic; the latter two are associated with more chronic antigen exposure and granulomatous inflammation. A more recent classification of HP partitions the disease into non-fibrotic and fibrotic forms based on the presence or absence of significant fibrosis on lung imaging (40).

HP may be confused with sarcoidosis. The presenting symptoms of HP include cough, dyspnea, fatigue and weight loss (40, 41) that are commonly found with sarcoidosis. Although both sarcoidosis and HP are associated with various radiographic features that are highly specific for each disease (Table 3), these features are often not present such that these diseases cannot be reliably distinguished on the basis of chest imaging findings. One potential distinguishing feature of sarcoidosis compared to HP is that sarcoidosis may involve extrapulmonary organs whereas HP is an isolated pulmonary disease. The diagnosis of HP is usually established if there is an appropriate temporal relationship to an exposure known to be associated with HP as well as clinical, serologic and/or radiographic findings that are consistent with the disease (40, 42). Suggestive serologic findings consistent with HP are the presence of specific serum antibodies to an antigen associated with HP. However, the presence of such antibodies without other clinical evidence is not diagnostic of HP, as the patient may be sensitized to the antigen without developing lung disease. Although the diagnosis of HP does not require biopsy confirmation when a specific causative exposure is suspected and serologic tests are available (40, 43–45), often this is not the case and a biopsy is performed. Although there may be an overlap between the biopsy features of sarcoidosis and HP, the histology of these two granulomatous diseases is somewhat different and may strongly favor one diagnosis over the other. Compared to sarcoidosis, the granulomas of HP are smaller, poorly formed and are not well-demarcated, are airway-centric and more commonly associated with multinucleated giant cells (30). Bronchoalveolar lavage (BAL) cell analysis has been advocated by some to differentiate sarcoidosis from HP on the basis of the percentage of lymphocytes or the ratio of CD4+/CD8+ lymphocytes. However, these BAL analyses have not been found to be adequately discriminatory (46, 47).

**Table 3:** Typical radiographic findings of pulmonary sarcoidosis.

**Radiographic features**	**Specific examples**
Micronodules in a peri-lymphatic distribution	Micronodules distributed along the bronchovascular bundle (Figure 1)
	Micronodules distributed in subpleural locations (Figure 2)
	Micronodules causing “beading” of the lung fissures (Figure 3)
Micronodules in the periphery coalescing into consolidated masses—“galaxy sign” (Figure 4)	
Relatively symmetric hilar lymphadenopathy with additional mediastinal lymphadenopathy	

### Chronic Beryllium Disease

**Figure 6:**
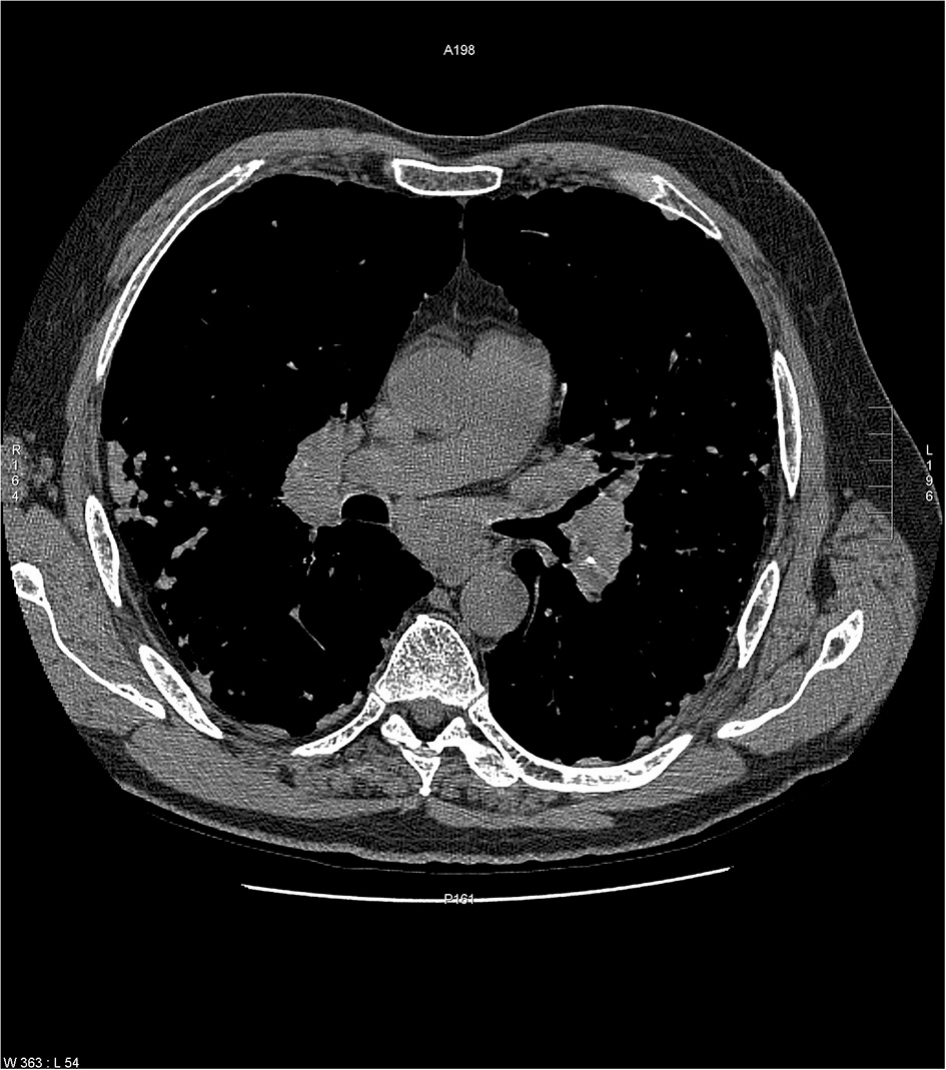
Chest radiograph of a patient with chronic beryllium disease showing imaging features typical of pulmonary sarcoidosis including bilateral hilar adenopathy with some calcium in lymph nodes. Reproduced with permission from Judson (52).

**Figure 7:**
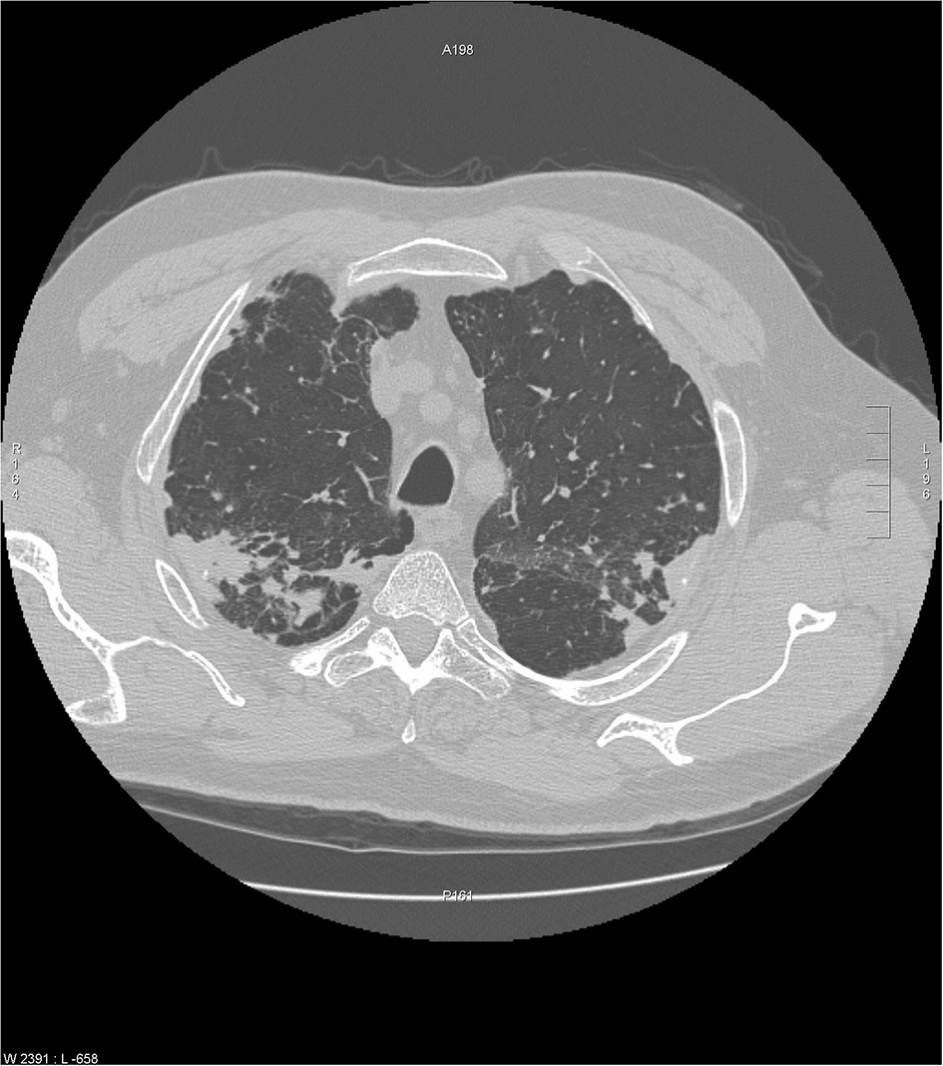
Another chest CT scan image from the same scan as Figure 6 showing peripheral and subpleural opacities. Reproduced with permission from Judson (52).

Chronic beryllium disease (CBD) is a granulomatous disease caused by exposure to beryllium. It may be indistinguishable from sarcoidosis both radiographically and histologically. CBD occurs in approximately 2 to 5 percent of beryllium exposed workers (48). Individuals who develop CBD are sensitized to beryllium by virtue of a specific mutation in the HLA-DPB1 gene, which is important for MCH class-II molecule function on antigen presenting cells. The latency from exposure to beryllium and the development of clinical CBD may vary from 3 months to 30 years (49, 50). CBD is primarily a granulomatous disease of the lung. Rarely, CBD may cause extrapulmonary granulomas, with skin being the most common extrathoracic site of disease followed by the liver and then disordered vitamin D metabolism leading to hypercalcemia and nephrolithiasis (51). The chest imaging findings of CBD mimic sarcoidosis both in terms of mediastinal adenopathy and the location of pulmonary micronodules (Figures 6, 7) (53). Histologic findings are identical to sarcoidosis with typical tight, well-formed non-caseating granulomas (54). CBD should be considered in any individual with chest imaging or a lung biopsy consistent with sarcoidosis who also has a history of beryllium exposure. The problem is that many CBD patients and their physicians are unaware of such exposures, and a significant percentage of CBD cases have been misdiagnosed as sarcoidosis (55). Beryllium exposure may occur in workers involved with several industries including dentistry, aerospace, aircraft production, nuclear power, ceramics, and engraving of gems (55). However, there are numerous other industries where beryllium exposure may be significant (56), and more cases of CBD have found to have been misdiagnosed sarcoidosis cases when a detailed occupational history for beryllium exposure has been elicited (55). The diagnosis of CBD is established when beryllium lymphocyte proliferation test (BeLPT) is performed on peripheral blood or bronchoalveolar lavage derived lymphocytes in a patient with a compatible radiographic or histologic picture of CBD. The BeLPT is considered positive when the lymphocytes are found to proliferate in a solution of beryllium sulfate (56). A similar granulomatous reaction has been reported with titanium and aluminum in case reports and small case series (57–59).

## Malignant Reactions

### Lymphoma

Lymphomas have been associated with granulomatous inflammation in lymph nodes and visceral organs that have been involved with lymphoma (60, 61). Granulomatous reactions are more common with Hodgkin than non-Hodgkin lymphomas (61–63), but they have been described with B-cell and T-cell non-Hodgkin lymphomas (64, 65). On occasion, lymphomas associated with granulomas have been misdiagnosed as sarcoidosis or tuberculosis (61). Hodgkin lymphoma can usually be diagnosed in these cases with careful histologic review that may reveal Reed-Sternberg cells. Analysis of lymphocyte markers within the granulomas and ancillary studies may also be useful in establishing the diagnosis of lymphoma (61). To complicate matters, sarcoidosis and lymphoma may occur in the same patient, and this association seems to be more common than by random chance (66, 67). The term, “sarcoid lymphoma syndrome” has been coined to reflect this association (68). It appears that either disease may occur first or both may occur concurrently (68).

### Sarcoidosis-Like Reactions of Malignancy

Sarcoid-like reactions have been described not only with lymphoma but with various solid organ malignancies (66, 69), including lung cancer (70, 71), breast cancer (72), colorectal (73), and genitourinary cancers (74). The granulomas are usually found in the vicinity of the tumor, either in the cancerous organ itself, draining lymph nodes, or adjacent to metastases (75). When granulomas are found in these locations in a patient with a known malignancy, a diagnosis of a sarcoidosis-like reaction of malignancy is suspect. Sarcoidosis-like reactions of malignancy are postulated to result from a T-cell mediated host response to soluble tumor antigens and products that may be shed by the tumor cells or released by tumor necrosis. Although the granulomas from sarcoid-like reactions contain B cell lymphocytes and sinus histocytes that are not typically observed in sarcoid granulomas (76–78), such an immunohistologic analysis is not performed clinically.

## Vasculitides/Connective Tissue Diseases

Because vasculitides are systemic disorders, commonly affect the lung and may be associated with granulomatous inflammation, they are occasionally confused with sarcoidosis. However, the clinical presentations and histologic findings of granulomatous vasculitides are usually sufficiently discordant from sarcoidosis that the two diseases can usually be easily differentiated.

Granulomatosis with polyangiitis (GPA), formerly known as Wegener's granulomatosis, is a vasculitis of small to medium-sized vessels that usually affects the upper airway, ear/nose/throat, airways, lung parenchyma and kidney. Common extrapulmonary presentations include otitis media, hearing loss, sinusitis, epistaxis, septal perforation, saddle nose deformity, hemoptysis and renal dysfunction (30). Although most of these presentations have been reported with sarcoidosis, they are relatively rare. Pulmonary involvement with GPA may result in cough, dyspnea, chest discomfort and pulmonary nodules and opacities on lung imaging that are all common presentations of pulmonary sarcoidosis. However, hemoptysis, alveolar hemorrhage and cavitary lung lesions are common presentations of GPA and are rare with sarcoidosis. Anti-neutrophil cytoplasmic antibodies vs. proteinase 3 (c-ANCA, PR3+) has a high specificity for active GPA (79). whereas these antibodies are routinely negative with sarcoidosis. Typical histologic features of GPA include necrotizing granulomas in combination with a necrotizing vasculitis (30), which are uncommon with sarcoidosis.

Eosinophilic granulomatosis with polyangiitis (EGPA), formerly known as Churg-Strauss syndrome is a vasculitis of small to medium-sized vessels that presents with the triad of asthma, eosinophilia and vasculitis (80). The asthma may predate the other manifestations of EGPA by several years (81). Wheezing is common with sarcoidosis, and it is often problematic to distinguish sarcoidosis from asthma (82). However, other common clinical manifestations of EGPA include palpable purpura, mononeuritis multiplex and glomerulonephritis that are uncommon manifestations of sarcoidosis. Eosinophilia may also occur in sarcoidosis but is rare (75), although one report suggested that it was common but not to the degree seen with EGPA (83). Chest imaging in EGPA often reveals patchy lung opacities. Perinuclear anti-cytoplasmic antibodies (p-ANCA) have a poor sensitivity for the diagnosis of EGPA, being positive in only 35–50% of cases (84). The diagnosis of EGPA is usually made based on clinical features, and these are usually adequately discordant from those of sarcoidosis that the two diseases can be distinguished. The disorders have clearly distinct pathology when a confirmatory biopsy is performed. The histologic features of EGPA include necrotizing granulomas in combination with a necrotizing vasculitis (30). Eosinophils are the predominant inflammatory cells in these lesions as opposed to neutrophils with GPA. Both the pathology of GPA and EGPA differ significantly from that of sarcoidosis, which almost never display a similar degree of necrosis and rarely is associated with a vasculitis.

Rheumatoid nodules are most commonly found subcutaneously at points of pressure (85), and may grossly appear similar to massive sarcoidosis skin and tenosynovitis lesions. Rheumatoid nodules may be distinguished from sarcoidosis in that the patients usually have a diagnosis of rheumatoid arthritis and have positive serology for that diagnosis. In addition, although rheumatoid nodules may demonstrate granulomatous inflammation histologically (86), other histologic features of these lesions are significantly different from sarcoidosis. These features include a central focus of necrosis that contains fibrinoid material, a surrounding region of palisaded macrophages and a marginal zone of vascular connective tissue often infiltrated with chronic inflammatory cells (85). Radiographically, rheumatoid lung nodules are usually cavitary (87) which is uncommon with pulmonary nodules associated with sarcoidosis.

## Localized Granulomatous Reactions To Foreign Substances Including Lung Aspiration

Innumerable foreign substances may penetrate the skin and induce a foreign body granulomatous reaction. These substances include particles used in tattoos and cosmetic fillers as well as substances that enter the skin from cutaneous trauma (88). In addition, numerous drugs may cause these reactions (88). These drugs may be systemic medications that may cause dermal deposits and subsequent granulomatous reactions, topical applications of drugs, or following injection of drugs of vaccines (88). Histology reveals granulomatous inflammation with epithelioid histiocytes with multinucleate giant cells. These giant cells are typically of a “foreign body type” with nuclei scattered irregularly throughout the cytoplasm, although Langhans cell giant cells may also be seen (89). In many cases, foreign substances may be recognized within these granulomas. These reactions are distinguished from sarcoidosis in that they are usually localized and often localized to the skin. In addition, there is usually a history of localized exposure to a foreign substance and/or a foreign substance is identified histologically within the granuloma.

Aspiration pneumonia may result in foreign body granulomas from aspirated material (36). Although a history of aspiration is usually present in cases of aspiration pneumonia, this is not always the case. Histologic examination may be necessary to confirm the presence of aspirated material within the granulomas or demonstrate other histologic features suggestive of necrotizing bronchopneumonia or organizing pneumonia (36).

## Drug-Induced Sarcoidosis-Like Reactions (DISRs)

A drug-induced sarcoidosis-like reaction (DISR) is a systemic granulomatous reaction that is indistinguishable from sarcoidosis and occurs in temporal relationship with initiation of an offending drug (90). DISRs represent a nearly perfect sarcoidosis mimic, and it is not clear if these drugs are truly causing sarcoidosis, rendering the immune system susceptible to the development of sarcoidosis, exacerbating subclinical cases of sarcoidosis, or are causing conditions that are distinct from sarcoidosis (90). There is no clinical presentation that distinguishes a DISR from sarcoidosis, and both have been associated with bilateral hilar adenopathy, uveitis, granulomatous inflammation of scars and tattoos, vitamin D dysregulation, elevated serum ACE levels, FDG uptake on PET scanning, and identical histology (90). Four common categories of drugs that have been associated with the development of a DISR are interferons, highly active anti-retroviral therapy, immune checkpoint inhibitors, and tumor necrosis factor alpha (TNF-α) antagonists (91–95). TNF-α antagonists are associated with DISRs even though some of them are effective therapies for sarcoidosis. Unlike sarcoidosis, DISRs often resolve after discontinuation of the offending agent, and may recur with re-challenge; these may be the only features that reliably distinguish these two entities. The diagnosis of a DISR requires the exclusion of alternative causes of granulomatous inflammation, the temporal relationship between a potentially causative drug and the onset of clinical features of sarcoidosis (that usually occurs 4 to 24 month after drug initiation) and the resolution of the sarcoidosis features after drug withdrawal (which does not always occur) (90). As these diagnostic criteria for a DISR are not completely distinct from sarcoidosis, many times these two conditions cannot be distinguished. Other than withdrawing the offending drug, a DISR is treated similarly as sarcoidosis. It may be best to continue the offending drug if it has been beneficial and add anti-sarcoidosis therapy. This is often the case with immune checkpoint inhibitor-induced DISRs, as it has been shown that immure-related adverse events (IRae's) to checkpoint inhibitors, of which a DISR is one, are associated with a potent anti-tumor response (96).

## Diffuse Granulomatous Reactions From An Autoimmune Inflammatory Syndrome Induced By Adjuvants

Diffuse granulomatous reactions to foreign substances differ from localized granulomatous foreign body reactions in that they are systemic and not localized to the site of the foreign material. Sarcoidosis like reactions may result from an autoimmune inflammatory syndrome induced by adjuvants (ASIA) (97, 98) that was previously described as “human adjuvant disease.” ASIAs when certain substances chronically stimulate immune pathways and prevent antigens from being degraded, thus prolonging antigen exposure to antigen presenting cells (98). Adjuvants that have induced ASIAs have included mineral oil (99), hyaluronic acid (100), and silicone (101). Several sarcoidosis like reactions related to ASIA have been reported after silicone breast implantation (102–104), and the sarcoidosis like reactions resolved after removal of the implant (105). ASIA-induced sarcoidosis syndromes appear indistinguishable from sarcoidosis and have presented with mediastinal lymphadenopathy (103, 106), Lofgrens syndrome (102), pulmonary nodules (102), uveitis (102), neurosarcoidosis (104), and skin lesions (103). ASIA-Induced sarcoidosis should be suspected when clinical features of sarcoidosis develop after exposure to a suspected adjuvant and may resolve if the foreign substance can be removed.

## Granulomatous Lesions of Unknown Significance (Glus Syndrome)

More than 30 years ago, a syndrome was described with prolonged fever; epithelioid granulomata in the liver, bone marrow, spleen, and lymph nodes; a benign course; and a tendency for recurrence. Unlike sarcoidosis, pulmonary granulomatous involvement is rare and usually confined to mediastinal lymph nodes (107). This entity has been labeled as the syndrome of granulomatous lesions of unknown significance (GLUS) (108, 109). Although it has been argued that the GLUS syndrome is a form of extrapulmonary sarcoidosis, differences include the following: (1) elevated serum angiotensin converting enzyme (SACE) levels have never been found with the GLUS syndrome, (2) the Kveim test has always been negative in the GLUS syndrome, (3) hypercalcemia is never found with the GLUS syndrome, and (4) immunotyping of lymphocytes within the granulomata of GLUS syndrome patients contain predominantly B-cells that is distinctly different from the granulomata of sarcoidosis patients that contain predominantly T-cells (107, 109). This syndrome is extremely rare, having been rarely reported during this millennium (107, 110).

## Granulomatous Interstitial Lung Disease (GLILD) Related To Common Variable Immunodeficiency (CVID)

**Figure 8:**
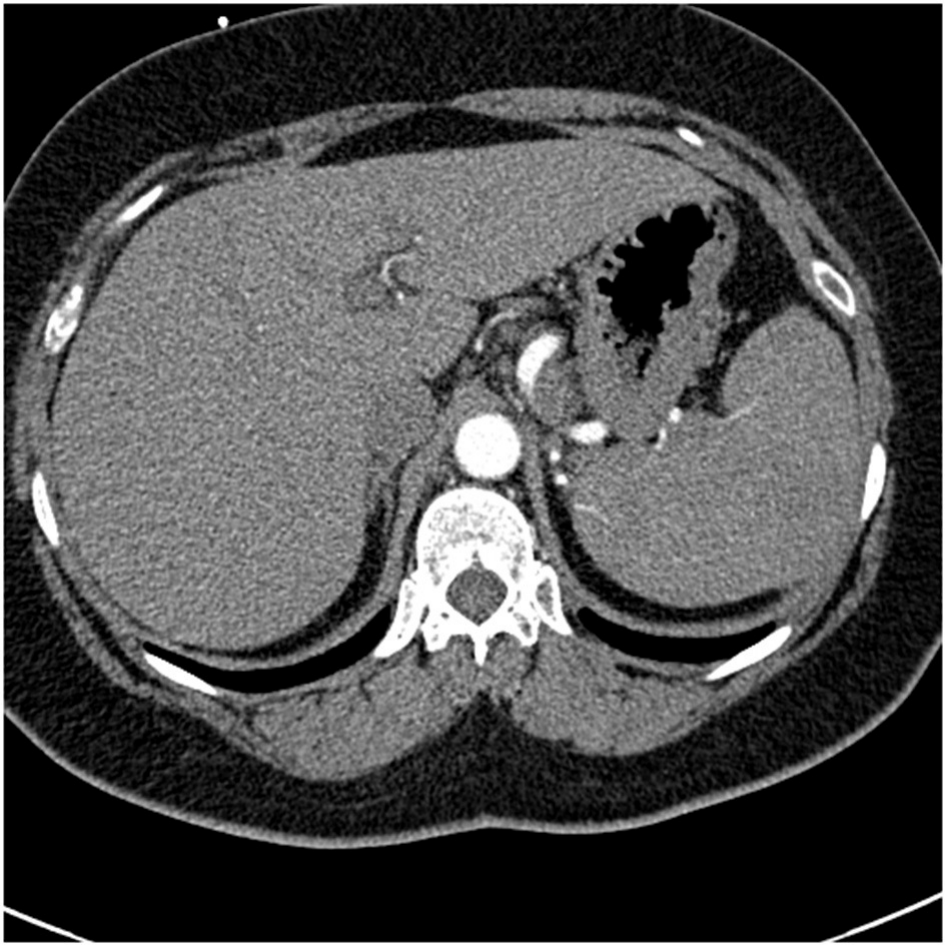
A lower chest CT scan image from the same scan as Figure 5 showing a mildly enlarged spleen, which is a common feature with common variable immunodeficiency.

Common variable immunodeficiency (CVID) is a primary immunodeficiency that may occur at any age and is characterized by an inability to mount specific antibody responses to ubiquitous exogenous antigens and low levels of IgG (111, 112). CVID patients are predisposed to develop sinopulmonary infections as well as malignancy (111). It is not surprising that radiographic manifestations of CVID would show findings consistent with chronic and/or previous lung infection such as bronchial wall thickening, bronchiectasis, ground glass opacities, scarring and fibrosis (113, 114). However, granulomatous pulmonary disease is found in some cases with or without lymphoproliferative features (lymphocytic interstitial pneumonia, follicular bronchiolitis, lymphoid hyperplasia, splenomegaly). The term granulomatous-lymphocytic interstitial lung disease (GLILD) has been used to describe this entity (112). The cause of this granulomatous inflammation is not currently known. Chest imaging in GLILD may show lung nodules and mediastinal lymphadenopathy, although there is often concomitant bronchiectasis and a tree-in-bud pattern (112, 115). As chest computed tomography (CT) is often used to assess the lungs, and the spleen is usually identified on such scans, splenomegaly is often seen with CVID/GLILD (Figure 8) (116). Pathology of GLILD lesions reveals non-caseating granulomas that are problematic to differentiate from sarcoidosis, although bronchiectasis, lymphocytic interstitial pneumonia, follicular bronchiolitis, and lymphoid hyperplasia may also be present. The BAL CD4/CD8 ratio is usually low in GLILD which may distinguish it from pulmonary sarcoidosis (117, 118).

## Necrotizing Sarcoid Granulomatosis

**Figure 9:**
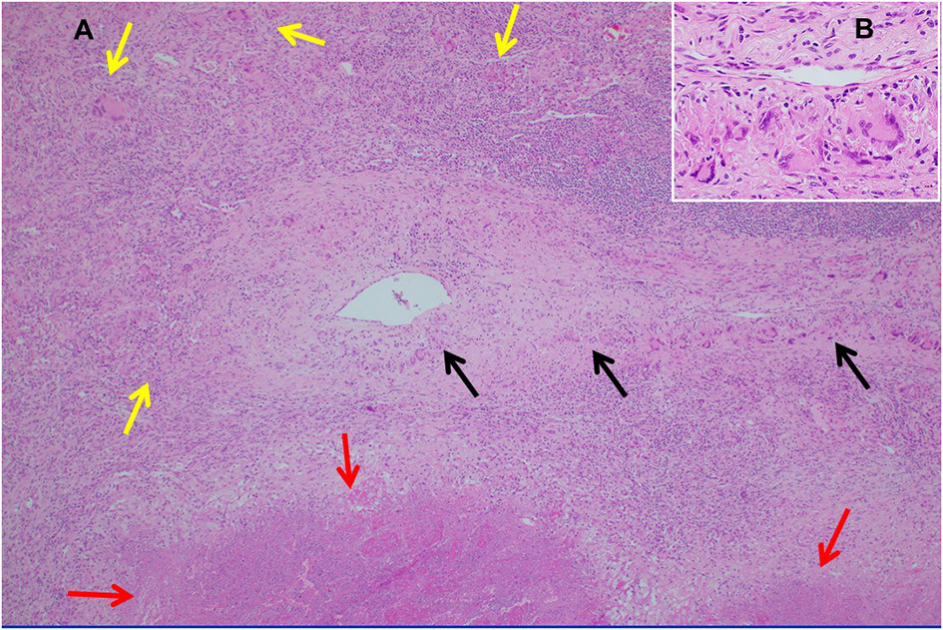
**(A)** Lung biopsy, necrotizing sarcoid granulomatosis. Low power image (H&E stain, 40x) of nodular replacement of a region of lung parenchyma by numerous non-necrotizing granulomas with admixed mild chronic inflammation, which is different in its appearance from classic nodular sarcoidosis by the presence of a broad area of parenchymal necrosis (red arrows) and prominent vascular involvement by non-necrotizing granulomas and chronic inflammation (black arrows). **(B)** Inserted higher power image of the pulmonary vessel from **(A)** with marked mural multinucleated giant cell rich inflammation distorting and compressing the vessel wall without associated vascular necrosis.

Necrotizing sarcoid granulomatosis (NSG) is a granulomatous disease consisting of granulomas that are often confluent, necrotic, and associated with a granulomatous and lymphocytic vasculitis (119). It is currently debated as to whether NSG is a form of sarcoidosis or a distinct disease entity (120). Pathologically, NSG is quite distinct from sarcoidosis in that significant necrosis is present and perivascular granulomas compress vessels without causing vascular necrosis (Figure 9) (120). The perivascular granulomas usually surround both arteries and veins (120, 121). Although sarcoidosis granulomas may show evidence of necrosis, necrosis is usually not a prominent feature whereas it is extensive with NSG; so much so that NSG often mimics the caseating granulomatous inflammation of tuberculosis except for the glaring difference that mycobacterial stains and cultures of NSG tissue are negative. NSG is strongly suggested by performing an elastin stain of involved tissue that demonstrates prominent granulomatous inflammation of the vasculature, which is unusual with sarcoidosis (120, 122). Radiographic presentations between NSG and sarcoidosis are somewhat different in terms of frequency, with mediastinal adenopathy being much less common with NSG, and large masses with or without cavitation more common with NSG than pulmonary sarcoidosis (120). Extrathoracic involvement with NSG in similar in frequency and organ distribution to sarcoidosis with the eye and skin being commonly involved. Elevations in serum ACE and serum IL-2 receptor are also common with NSG (120, 123).

## BLAU Syndrome

Blau syndrome is a granulomatous disease affecting almost exclusively the skin, joints, and/or uveal tract of the eye (124). The disease has an autosomal dominant pattern of inheritance and usually presents before age 4 (125, 126). The disease rarely occurs outside of the usual triad or organs such as in the CNS, skin, and extremely rarely, the lung (124, 127). Blau syndrome is a genetic disease caused by mutations in the NOD2 gene (128). The granulomas appear identical to those of sarcoidosis and are usually tight and non-caseating (124, 126, 127).

## Orofacial Granulomatosis

Orofacial granulomatosis (OFG) is often used as a term to describe all conditions with oropharyngeal swelling and histologic evidence of granulomatous inflammation (129). The lips are most commonly affected although any orofacial areas may be involved (129–131). The clinical manifestations of OFG range from subtle mucosal swelling of the lips and face to oral ulcerartions and neurologic manifestations of the head and neck (132–134). Melkersson-Rosenthal syndrome is a form of OFG consisting of the triad of orofacial edema, facial palsy, and a fissured tongue (lingua plicata) (131). Miescher's chelitis is a specific form of OFG where the sole manifestation is labial swelling from granulomatous inflammation. Both Melkersson-Rosenthal syndrome and Miescher's chelitis are thought to be idiopathic forms of OFG and distinct from sarcoidosis and other secondary forms of OFG in that they are not systemic diseases and have characteristic clinical manifestations (129). Histopathologic features of OFG include non-caseating granulomas, ectatic lymphatic vessels, tissue edema, and perivascular lymphohistiocytic infiltrates (133, 135, 136). Although sarcoidosis is one cause of secondary OFG, it is a relatively rare one. In one series of 104 patients with OFG, 3% had sarcoidosis. The most common diagnoses in this series were idiopathic (including Melkersson-Rosenthal syndrome and Miescher's chelitis) 41%, roseacea/acne vulgaris 12%, Chron's disease 10%, and contact dermatitis 8% (129). All these conditions should be searched for in an OFG patient.

## Crohn's Disease

Sarcoidosis and Crohn's disease are granulomatous disorders with overlapping features. Distinguishing these two entities may be problematic. Common presentations of Crohn's disease include bloody and non-bloody diarrhea. As gastrointestinal tract sarcoidosis usually causes no symptoms (137), the diseases can usually be distinguished on that basis. In particular, rectal lesions, perianal lesions and fistulas in any location strongly favor a diagnosis of Crohn's disease rather than sarcoidosis. Intestinal Crohn's disease may cause anorexia, loose or frequent stools, fever, night sweats and weight loss. All of these symptoms may be present with intestinal sarcoidosis, although this form of sarcoidosis is rare (138). Granulomas have been found in 15 to 70 percent of patients with Crohn's disease (139). Although the histologic features of granulomas in sarcoidosis and Crohn's disease usually differ, with sarcoidosis granulomas typically being much more well-formed, there is significant overlap such that the two diseases cannot be reliably distinguished. The presence of crypt inflammation, apthae, and ulcers are more suggestive of Crohn's disease (137). The lack of granulomas on biopsy would suggest Crohn's disease over sarcoidosis. In addition, Crohn's disease often causes transmural inflammation whereas intestinal sarcoidosis usually involves only the mucosa (137, 140). Extraintestinal manifestations of Crohn's disease do occur and some of these are similar to systemic manifestations of sarcoidosis. Although granulomatous pulmonary nodules have been described with Crohn's disease (141–143), this rarely occurs. Table 4 displays clinical data that may distinguish Crohn's disease from sarcoidosis.

**Table 4:** Clinical data supporting the possibility of sarcoidosis vs. Crohn's disease.

	**Supports sarcoidosis**	**Supports Crohn's disease**	**Does not favor either diagnosis**
**Clinical presentation**			
Isolated GI tract disease		++	
History of extra GI tract sarcoidosis	++		
GI symptoms		++	
Rectal/perianal lesions		+	
Disease isolated above the ligament of Trietz	+		
**Histology**			
Granulomas			√
Crypt inflammation		++	
Aphthae		++	
Ulceration		++	
**Extra GI tract manifestations**			
Erythema nodosum			√
Pyoderma gangranosum		++	
Aphthous ulcers		++	
Cheilitis		++	
Uveitis			√
Arthritis			√
Pulmonary nodules and/or thoracic adenopathy	++		
Spondylitis		++	
Uric acid nephrolithiasis		+	
Calcium oxalate nephrolithiasis			√
Elevated 24 h urine calcium or elevated serum 1,25 di-hydroxy vitamin D	++		

*+: supports diagnosis ++: strongly supports diagnosis √: condition common in both disorders; Patel et al. (137), reproduced with permission (Practical Gastroenterology)*.

## Primary Biliary Cirrhosis

Sarcoidosis may be confused both clinically and histologically with primary biliary cirrhosis. The granulomas of sarcoidosis are often situated in the portal triads resulting in bile duct obstruction (144). This may result in an obstructive hepatopathy with pruritus that is common with primary biliary cirrhosis (145). Granulomatous inflammation can be seen with both conditions (145). Differentiating features include that primary biliary cirrhosis rarely causes pulmonary symptoms or any extrahepatic symptoms (145). In addition, the anti-mitochondrial antibody (AMA) is positive in approximately 90% of primary biliary cirrhosis cases. However, on rare occasions AMA is positive with sarcoidosis (146, 147). Supposedly, the M2 form of the AMA is specific for primary biliary cirrhosis and should distinguish this disease from sarcoidosis.

## Summary

Although sarcoidosis is referred to as a systemic granulomatous disease of unknown cause, sarcoidosis obviously has a cause. In the future, once the cause of sarcoidosis is determined, it may become less problematic to distinguish sarcoidosis from its granulomatous mimics. At that point, we may deduce that several mimics of sarcoidosis are actually forms of sarcoidosis. Until that time when the immunopathogenesis of sarcoidosis becomes clear, we must rely on our understanding of the clinical features of sarcoidosis and alternative granulomatous diseases to distinguish these entities. Such a distinction requires understanding of the medical history, demographics, potential exposures, radiographic manifestations, histologic features, and laboratory findings associated with these disorders. Distinguishing sarcoidosis from its mimics is not an academic exercise, as minimizing the misdiagnosis of these diseases will result in improved patient care and less patient suffering.

## Data Availability Statement

The raw data supporting the conclusions of this article will be made available by the authors, without undue reservation.

## Author Contributions

The author confirms being the sole contributor of this work and has approved it for publication.

## Conflict of Interest

The author declares that the research was conducted in the absence of any commercial or financial relationships that could be construed as a potential conflict of interest.
